# Oscillations and Spike Entrainment

**DOI:** 10.12688/f1000research.16451.1

**Published:** 2018-12-20

**Authors:** Charles J. Wilson, Matthew H. Higgs, DeNard V. Simmons, Juan C. Morales

**Affiliations:** 1 Department of Biology, University of Texas at San Antonio, San Antonio, TX, 78249, USA

**Keywords:** LFP, oscillation, phase-locking

## Abstract

Oscillatory input to networks, as indicated by field potentials, must entrain neuronal firing to be a causal agent in brain activity. Even when the oscillatory input is prominent, entrainment of firing is not a foregone conclusion but depends on the intrinsic dynamics of the postsynaptic neurons, including cell type-specific resonances, and background firing rates. Within any local network of neurons, only a subset of neurons may have their firing entrained by an oscillating synaptic input, and oscillations of different frequency may engage separate subsets of neurons.

## Field potential oscillations

Oscillatory field potentials are ubiquitous, being seen in nearly all parts of the brain and at all scales of locality from the EEG to local circuits. The study of these oscillations has been a major theme in basic and clinical neurophysiology since the discovery of the EEG by Berger (see review in 1). Field potential oscillations span a range of frequencies, and there can be multiple prominent oscillation frequencies in the same place at any one time. The spectral components of field potentials are separated by spectral analysis, which treats the signal as consisting entirely of oscillations over a range of frequencies. The most prominent frequencies are seen as peaks in the resulting spectrum. The enormous body of literature from these studies contains a trove of correlations between oscillatory field potentials and behavioral and experimental states of all kinds, including important practical correlations with brain diseases (for a review, see 2). Frequency components of the field potentials recorded across structures may be out of phase (asynchronous) or in phase (synchronous), and synchronously oscillating brain regions are often interpreted as being more effectively connected than asynchronously oscillating ones, a notion sometimes called “communication through coherence” ^3^. In this view, field potentials are taken as an indicator of patterning in the population activity of brain structures and coherent field potentials are taken as indicators of shared or reciprocally generated population activity.

## Why care about field potentials?

Although there is little controversy about the existence of oscillatory field potentials, there is much less consensus on their interpretation. The appearance of a field potential oscillation does not imply any particular circuit or cellular mechanism. Are the oscillations meaningful signals that we can interpret, or are they epiphenomena of synaptic transmission and neuronal circuit interactions?

Neurons normally do not communicate directly via field potentials. There are some exceptions ^4^, but most field potentials are consequences, not causes, of neuron communication (for example, 5). Currents running longitudinally within dendrites and axons produce extracellular currents as their return path, and these produce a local field potential (LFP) that can be recorded from an intracerebral microelectrode. Currents from all parts of all cells near an LFP electrode are averaged in the field. Which frequency components survive this interaction to be visible to the extracellular electrode depends on details of timing and on the geometric arrangements of dendrites and axons of neurons receiving the synaptic inputs. Usually, it is not certain just how local an LFP really is. This must be resolved independently in each brain region, and care must be taken to avoid contamination by strong current loops generated in distant structures ^6^. Intracellular currents produced by subthreshold voltage-sensitive ionic conductances and action potentials also have an extracellular component and contribute to the field potential (for example, 7). Because of their composite origin, it cannot be concluded that oscillations prominent in field potentials signal correspondingly large changes in neuronal membrane potentials. Synaptic currents that are asynchronous among neurons may produce much larger responses in neurons but fail to summate in the LFP (for example, 8), and periodic synaptic responses of neurons need not correspond in phase or frequency with the LFP ^9^. But most of all, field potentials are not propagated. They do not carry information from one part of the brain to the next (for example, 10). Given the unavoidable uncertainty about the origin and meaning of field potentials, why should we place so much importance on the spectral composition and relative phases of their oscillations?

## Spike-field entrainment

LFP oscillations can inform us about communication between brain structures to the extent that they predict the pattern of action potentials in neurons whose axons carry signals from one brain region to the next. In some brain structures, the majority of synapses are from neurons located within the local circuit or from neurons located in a reciprocally connected synaptic target. In the first case, an LFP oscillation may reflect local firing synchrony, producing coherence of locally generated synaptic currents. In the second, LFP coherence may indicate that neurons in the two regions are synchronized, but action potentials remain the obligatory intermediate in their communication ^11^. There have been many studies of the timing of action potentials relative to LFP oscillations. A single-unit recording and the corresponding LFP can be recorded from the same electrode. The two are mostly but not completely separable ^12, 13^ because extracellularly recorded action potentials contain higher frequencies (more than 500 Hz) than the local field (about 1 to 200 Hz). The firing of a neuron is said to be related to an LFP oscillation if spikes preferentially occur at one or more specific phases of the oscillation.

If the LFP waveform is sufficiently close to a single sinusoid, the assignment of phase is straightforward. However, most field potentials are a combination of oscillations over a continuous range of frequencies and so are not simply sinusoidal. Spiking may be closely related to the LFP for some oscillation frequencies and not others, so a method for separating out specific LFP frequency components is required for spike-field studies ^14^ (see “Cellular resonance” section below).

## Entrainment is specific for cell types

Different kinds of neurons receive different inputs, so only a subset of cells need receive synapses from an oscillatory input reflected in the LFP. Synaptic connectivity often varies among neurons of different cell types, so it is not surprising that entrainment to specific frequency components in the LFP is cell type specific. However, it is not usually known whether cell type differences in entrainment are caused by differences in connectivity, differences in postsynaptic cell properties, or something else. Whatever the causes may be, cell type differences in entrainment are common. In the hippocampus, for example, nearly all neurons show a preference to fire at some phase of the theta oscillation, but there are cell type differences in entrainment to the gamma oscillation. Firing in the bistratified interneuron is strongly locked to the gamma oscillation, whereas O-LM interneuron firing is only weakly entrained ^15, 16^. Hippocampal neurons of different types entrained by the theta oscillation differ in preferred firing phase ^17^. In the orbitofrontal cortex, cells preferentially align their firing with the theta or gamma oscillation but not both ^18^. In the striatum, firing of fast-spiking interneurons is often strongly locked to LFP oscillations in the gamma range whereas other cell types fire mostly in relation to lower-frequency components ^19– 21^. In the visual cortex, spikes of somatostatin neurons are most closely phase-aligned on lower-frequency oscillations (5 to 30 Hz) compared with parvalbumin-positive fast-spiking neurons, whose spikes are aligned to higher (20 to 80 Hz) LFP components ^22^. Although some of these cell-specific differences in the frequency-selectivity of entrainment probably arise from differences in connectivity, some are certainly caused by intrinsic frequency preferences.

## Cellular resonance

The same methods used to measure the entrainment of spikes to the LFP can be used to measure the intrinsic frequency-sensitivity of a cell but with a key difference. The input used to measure a cell’s intrinsic frequency-sensitivity is controlled by the experimenter, so frequencies can be represented more equitably than they are in nature. A broadband noise waveform or an artificial synaptic current barrage contains oscillations of equal amplitude at many frequencies. By injecting a broadband current into neurons and calculating the spectrum of spike entrainment, the frequency preferences of neurons can be measured and compared with the spectrum of spike-field entrainment. Inputs from other neurons are not involved because the stimulus is injected directly into the single cell. When this is done, neurons of different types produce very different spectra (for example, 23). Some cells (for example, the parvalbumin-containing fast-spiking interneurons in the striatum) have an entrainment maximum somewhere in the range of gamma oscillations (30 Hz and higher). Another interneuron in the same structure, the cholinergic interneuron, has an entrainment maximum closer to 1 Hz. In contrast, the principal neuron of the striatum, the spiny projection neuron, is specifically entrained at frequencies near its own firing rate at the time of measurement and its preference changes when the cell’s firing rate changes ^23, 24^.

**Figure 1.**
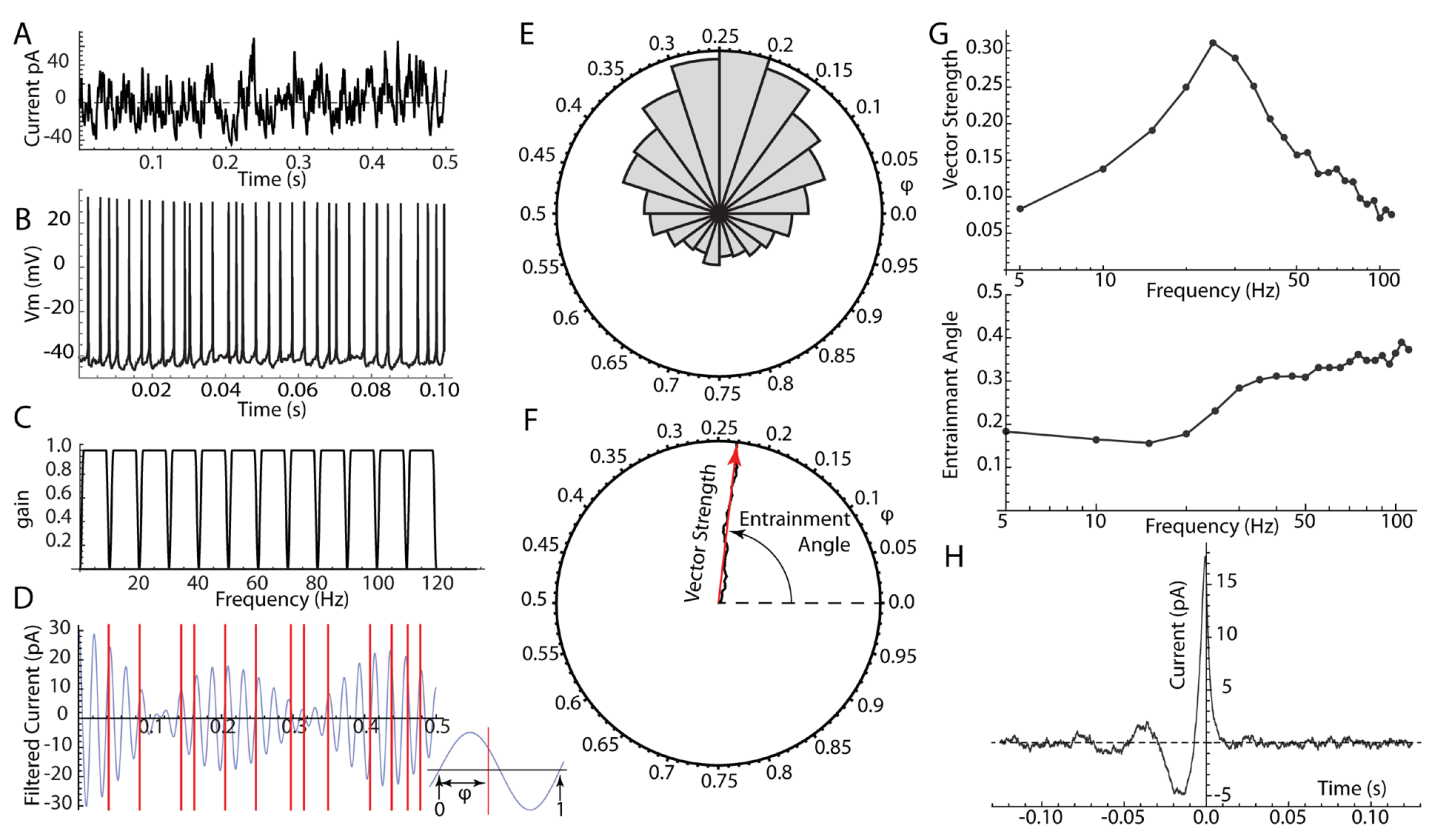
Measuring entrainment. ( **A**) Broadband noise current stimulus applied to the cell via intracellular (perforated patch) recording. ( **B**) Membrane potential responses and spiking in response to the broadband stimulus. ( **C**) Series of bandpass filters used to separate frequency components of the current stimulus. ( **D**) One of the filtered input frequency components (25 Hz; blue waveform) and spike times (red lines). Inset: Measurement of spike phase (φ) from the filtered frequency component. ( **E**) Phase histogram for spikes measured on the 25 Hz frequency component. ( **F**) Normalized vector sum of all the spike phases from the histogram in **E**. Length of the result is the vector strength, and the angle of the resultant vector is the entrainment angle. ( **G**) Vector strength (upper) and entrainment angle (lower) for each of the frequency components obtained using the filter set shown in **C**. ( **H**) Spike-triggered average of the same data used in **A**– **G**. Note oscillation at about 25 Hz, corresponding to the peak in the entrainment spectrum shown in **G**.

All methods for detecting frequency-specific spike entrainment are fundamentally similar. The injected current (or LFP) is filtered by using a series of bandpass filters to extract a set of nearly sinusoidal component waveforms, each centered on a single frequency. The phases of action potentials are readily measured relative to each of these frequency components ( Figure 1). If the cell is insensitive to input at one of the frequencies, the distribution of phases will be circularly uniform. All measures of entrainment rely on the deviation of the phase distribution from uniformity. If the phase distribution is unimodal, a vector sum is a suitable measure. Each spike phase is expressed as a unit vector, and these are summed and normalized by the number of spikes. The length of the resultant vector sum (vector strength) is a measure of the strength of coherence between spikes and the input oscillation. The angle of the resultant vector (entrainment angle) is the average phase of the neuron’s firing with respect to that frequency. The vector strength and entrainment angle at each frequency make up a spectrum of the neuron’s frequency preference and a spectrum of its preferred phase for each frequency ( Figure 1E, F). These measures can be misleading for input frequency components slower than the cell’s firing rate. For example, if a cell was 2:1 phase-locked (so that it fired precisely at two phases on every cycle of the oscillation), the vector strength would badly underestimate the strength of entrainment and the measured entrainment angle would be a phase at which the cell never fired. A more general approach is construction of a phase histogram showing the probability of firing at all phases of any one LFP frequency component ( Figure 1E). There are a variety of ways to measure deviation from the uniform distribution. The uniform distribution is the maximum-entropy circular distribution, making distribution entropy a good assumption-free measure. For kinds of entrainment with known phase distribution shapes, model-based methods may be most powerful ^25^.

An alternative class of methods for measuring frequency-specific spike entrainment is based on the spike-triggered average (for example, 26, 27). This is the converse of the spike-phase methods. The spike-triggered average is calculated by aligning the LFP to each action potential and averaging the result, producing an average of the LFP over a range of times (at least one cycle of the oscillation) around the spike. If firing were independent of the field potential, the average would be flat. If the cell is entrained to one or more frequency components in the field potential, those frequencies will be prominent in the average. Fourier transformation of the spike-triggered average produces spectra of component magnitudes and phases like those obtained by the spike-phase method. Spike-triggered averaging is more efficient to compute and more easily implemented, but calculating the statistical significance of frequency-specific entrainment is more difficult with this method. As with the vector sum method, spike-triggered averages are most simply interpreted when the cell’s firing rate is no faster than the frequency of the relevant LFP oscillation.

## Membrane impedance resonance

What causes cell type- and frequency-specific entrainment, and why do some cells show a fixed frequency preference whereas others shift their entrainment frequency with firing rate? Possibly, some of the answer can be found in the subthreshold resonance of cell membranes.

**Figure 2.**
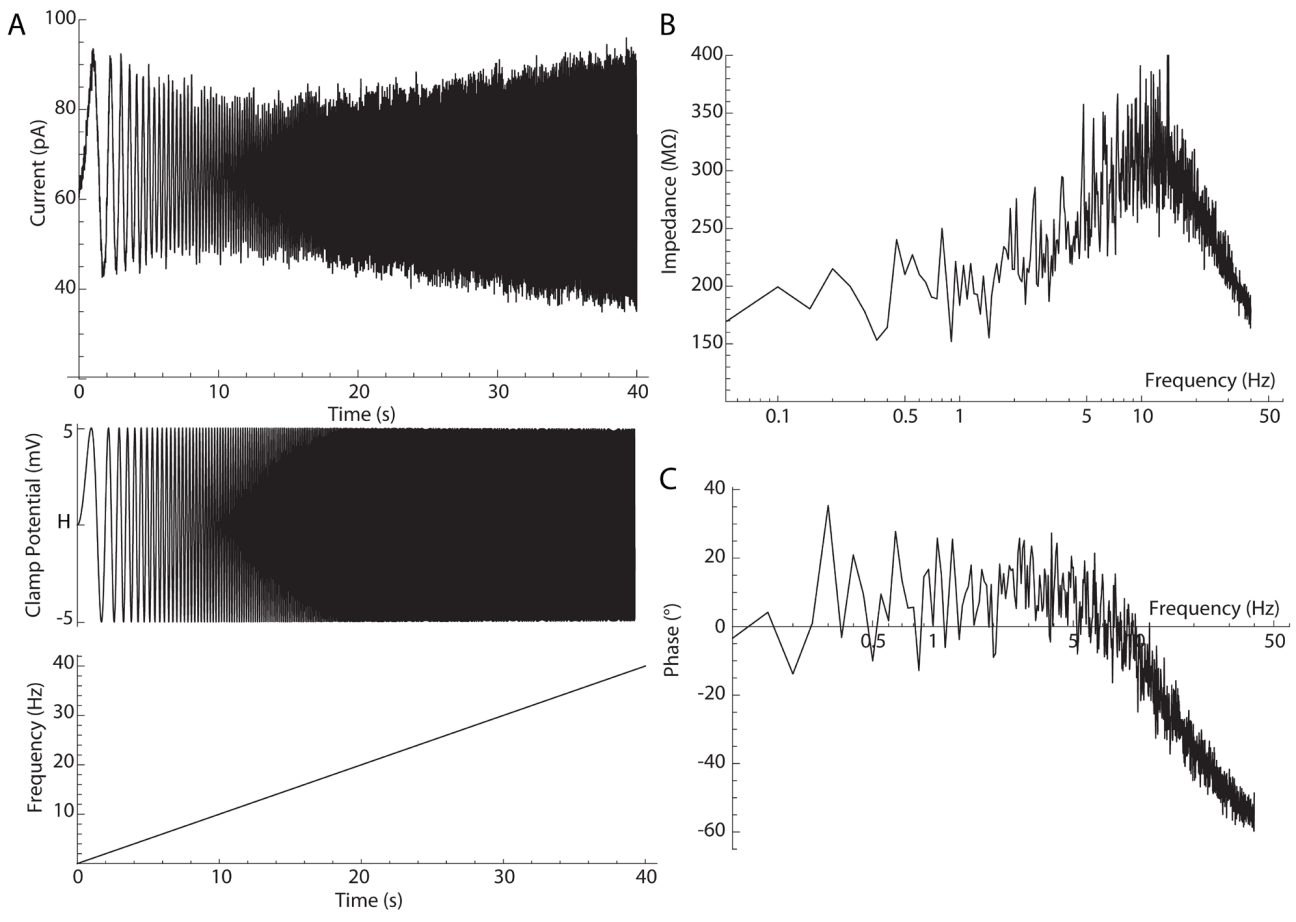
Measuring membrane resonance. ( **A**) Voltage clamp protocol for measuring resonance. The neuron’s voltage is clamped to a sine wave voltage chirp (middle) centered on a holding potential ( **H**). The frequency of the sine wave is increased linearly from 0 to 40 Hz over 40 seconds (bottom). The amplitude of the resulting sinusoidal clamp current (top) is inversely proportional to the membrane impedance. The minimum current amplitude around 11 s indicates the resonant frequency for the cell’s subthreshold impedance. ( **B**) Impedance, measured as the ratio of voltage command sine wave amplitude to clamp current, over the range of frequencies in the voltage command chirp. The impedance peaks at a resonant frequency near 11 Hz. ( **C**) The phase angle (angle between the voltage command and the current) at each frequency. At low frequencies, the voltage leads the current, whereas at high frequencies beyond the cell’s resonant frequency, the voltage lags behind the current. The frequency of zero phase difference occurs near but slightly below the frequency of peak resonance.

Not all cells show membrane resonance. When resonance is absent, the membrane potential response is maximal for constant input current and decreases for sine wave currents of increasing frequency. In all cells, high-frequency input currents are effectively shunted by the membrane capacitance, and in non-resonant cells, this is a primary determinant of membrane frequency response. In cells that have membrane resonance, the membrane response to an oscillating injected current first increases with frequency, then peaks at a non-zero frequency, and declines at higher frequencies ^28, 29^. Membrane resonance is caused by voltage-dependent ion channels activated in the subthreshold membrane potential range. Specifically, resonance requires ion channels producing currents that oppose voltage changes but with a delay. These channels, called resonant or restorative channels, interact with the cell membrane capacitance and with channels that amplify the voltage responses (amplifying or regenerative channels) to produce resonance in the membrane voltage response at subthreshold or near-threshold voltages. The influence of membrane resonance can be detected as a frequency-dependent increase in the voltage response to a small oscillating current when the cell is not firing or can be seen in voltage clamp as a decrease in the current required to impose an oscillating voltage ^23, 30^ ( Figure 2). Cells with strong membrane resonance also show subthreshold membrane potential oscillations following transient perturbations that do not trigger action potentials. In such cells, a small amount of noise is sufficient to maintain a constant small oscillation that can be seen on the membrane potential when the cell is not firing.

## Spiking in neurons with membrane impedance resonance

Membrane resonance is measured in the absence of action potentials. Once a cell begins to fire, there is no guarantee that membrane resonance will be influential enough to impose a preferred frequency for spike entrainment. Different and larger currents triggered in the course of firing may take control of membrane dynamics and overshadow the subthreshold currents. In the special case when a resonant cell is below (but close to) its firing level and an oscillating input of an appropriate size is applied, firing can occur specifically on the peaks of the driven oscillation. Because the size of the membrane potential oscillation is maximal at the resonant frequency, spikes can be made to occur specifically in the frequency range of resonance. However, this is a fragile situation because it relies on careful selection of the background current and just the right amount of oscillating input. If either of these is reduced, there is no firing at all. If the background level of excitation is a little greater, the cell may fire repetitively even in the absence of the oscillating input.

Once a cell begins to fire repetitively, everything is changed. Action potentials activate ion channels that are not normally engaged in the subthreshold range. Many of the currents generated outlast the action potential and can produce both reductions of excitability (refractory period) and increases of excitability (supernormal periods) during the interval between spikes ^31^. When the period of an oscillating stimulus aligns with the supernormal period, such cells will phase-lock their spiking to the stimulus. Like subthreshold resonance, this is a fixed frequency preference, set by the time course of spike-triggered ion channel activation and inactivation. In some neurons, the frequencies of subthreshold resonance and spike-driven resonance may be nearly the same. Neurons with post-spike oscillations of excitability generated by the same ion channels as their subthreshold resonance produce very strong spiking resonance at nearly (but not necessarily exactly) the same frequency as their subthreshold resonance. The squid axon (in low Ca ^2+^ concentration) and the original Hodgkin–Huxley mathematical model of the squid axon behave like this ^32, 33^ and they show very powerful resonance at a fixed frequency. Some fast-spiking interneurons show a similar combined subthreshold and spiking resonance at a fixed frequency ^23, 34^. However, in most neurons, there are spike-triggered ion channels (for example, calcium-dependent potassium channels) that are not active in the subthreshold membrane potential range, and these may reduce or abolish the effect of subthreshold resonance during repetitive firing.

It is possible to construct cell models with subthreshold resonance but no spiking resonance generated by post-spike refractory or supernormal periods. This is done by omitting spike-triggered currents from the model entirely, replacing them with a simple voltage threshold and post-spike voltage reset. Although there are probably no real neurons like these models, they allow us to visualize the effect that pure subthreshold resonance would have on repetitive firing. In these models, the influence of subthreshold membrane resonance can still be detected during repetitive firing, at least when the firing rate is less than the subthreshold resonant frequency ^29, 35, 36^.

## Entrainment of neurons with no membrane impedance resonance

Many neurons are non-resonant, meaning that they have neither a supranormal period after spiking nor subthreshold membrane resonance. Spike-triggered currents in non-resonant neurons produce only net decreases in excitability (refractoriness). These neurons may oscillate anyway, either because they have non-inactivating depolarizing currents that drive spontaneous activity or because they are tonically depolarized by synaptic currents. The rate of their ongoing firing is determined by both the strength of the depolarizing current driving their activity and the rate at which refractoriness decays after each action potential.

**Figure 3.**
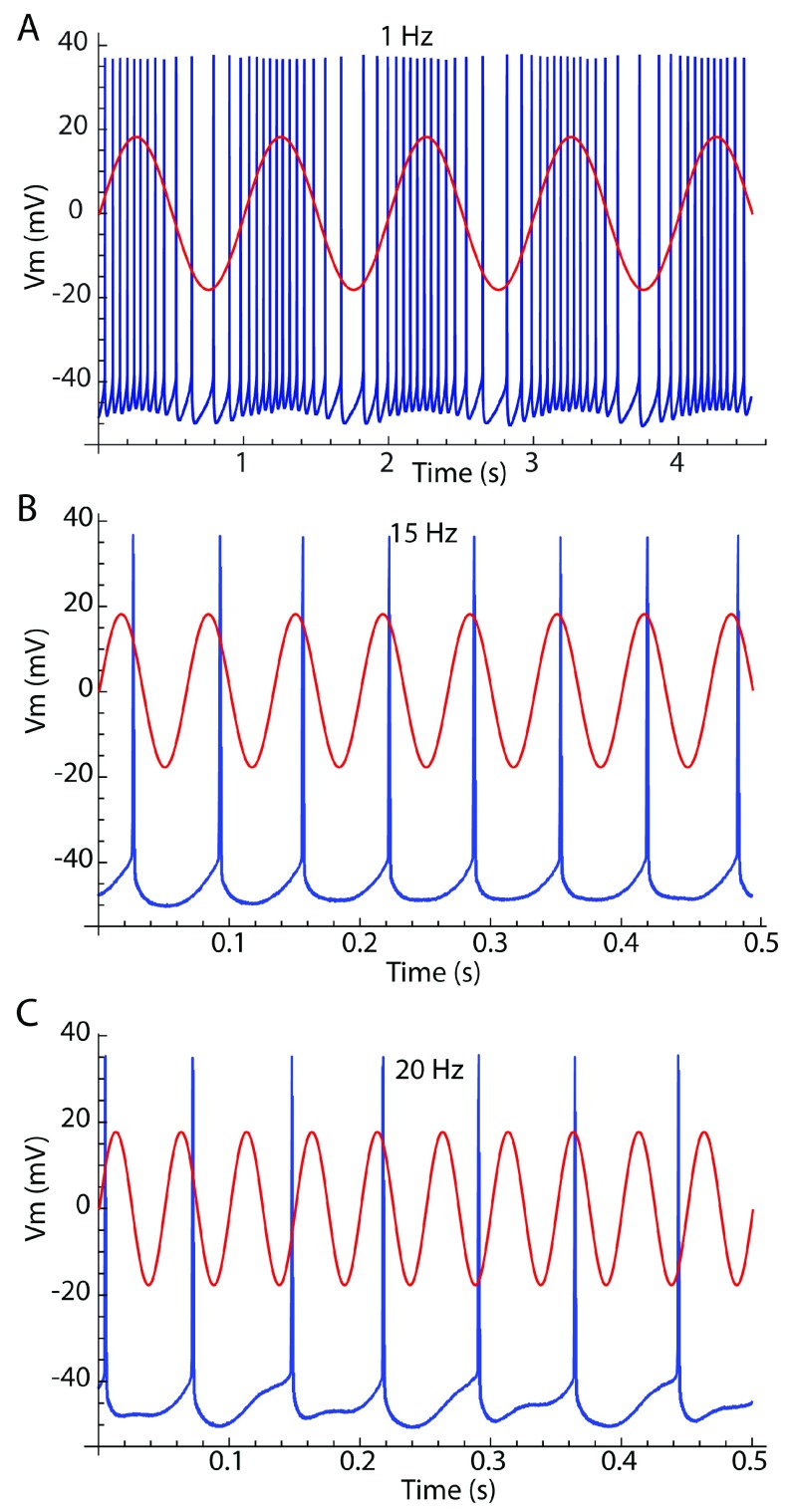
Entrainment modes. Neuronal membrane potential is shown in blue, and stimulus current waveform is overlaid in red. ( **A**) Rate modulation occurs when the cell’s firing rate is faster than the stimulus frequency. The cell’s background firing rate is 15 spikes/s. The stimulus frequency is 1 Hz. ( **B**) One-to-one phase-lock occurs when the stimulus frequency is near the cell’s firing rate. Stimulus frequency is 15 Hz. ( **C**) Irregular firing occurs when the stimulus frequency and the cell firing rate are not commensurable.

An oscillating input may entrain a non-resonant cell, either by modulating its rate of firing or by controlling the timing of its action potentials without altering their rate ^24, 37– 40^ ( Figure 3). For stimulus oscillation frequencies below the cell’s baseline (unperturbed) rate, the cell may fire several action potentials on each stimulus cycle. At such low stimulus frequencies, action potentials may not be phase-locked to any one phase on the signal waveform, but they are restricted mostly to the depolarizing half of the stimulus cycle. Slow oscillatory input may not alter the firing rate of a cell averaged over the stimulus cycle, but during the depolarizing phase of the oscillating input the cell’s instantaneous firing rate can increase substantially. This produces a periodic bursty pattern, in which bursts are entrained to the stimulus.

At stimulus frequencies greater than half the cell’s baseline firing rate, cells may fire spikes individually entrained in a repeating sequence of stimulus phases ^24^. Oscillating inputs sufficiently close to the cell’s baseline rate will elicit 1:1 phase-locking, meaning that the cell fires one spike at a single phase on each cycle of the input. To achieve phase-locking, the cell must usually change its rate slightly to match the stimulus. The range of frequencies over which the cell will adjust its firing rate to maintain phase-lock depends on the amplitude of the oscillating input. Large inputs produce phase-locking over a wide range of frequencies. The phase at which the cell locks varies continuously with changes in stimulus frequency within this range. At some stimulus frequency, the cell fires at the peak of the oscillatory current. This has been called the “preferred frequency” ^41^ or the “phasonance frequency” ^36^. At stimulus frequencies below that, spiking occurs before the peak of the stimulus current, whereas at higher frequencies phase-lock is later and can even occur during the hyperpolarizing phase of the stimulus ^24^. When phase-locked, the timing of spikes is highly reliable and noise-insensitive ^24, 40^. At higher frequencies still, phase-lock fails and cells again fire in a more irregular and noise-sensitive pattern. Neurons may become phase-locked again at stimulus frequencies twice their unperturbed firing rate and at even higher harmonics. At these frequencies, the cell skips cycles of the stimulus, and the number of skips is determined by the cell’s unperturbed firing rate.

**Figure 4.**
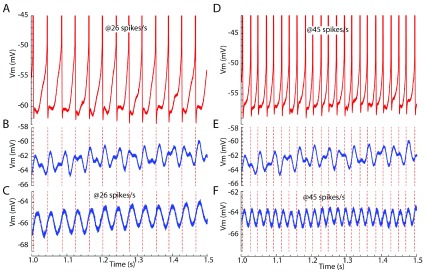
Frequency-dependent spike entrainment in a non-resonant striatal spiny neuron. Two different frequencies of sinusoidal current (26 and 45 Hz) were injected intracellularly while the cell was firing at one of two frequencies: 26 or 45 spikes/s. The firing times are compared with the membrane voltage waveform when the cell was hyperpolarized slightly to prevent firing and receiving the same two sinusoids or each of the sinusoidal currents was presented alone. ( **A**) Firing at 26 spikes/s while stimulated with both sine waves. ( **B**) The subthreshold membrane potential waveform for the same cell with the same stimulus but hyperpolarized slightly to prevent spikes. Note that the action potentials shown in A do not align on the voltage peak or any other consistent feature of the membrane potential waveform. ( **C**) The subthreshold membrane potential response to the 26 Hz sinusoidal current presented alone. Note that spikes generated in the presence of both frequencies are phase-locked to the 26 Hz sine wave component, regardless of the presence of the 45 Hz sine wave. ( **D**) The same neuron in the presence of the same stimulus shown in A but now firing at 45 spikes/s. ( **E**) Spike timing at 45 spikes/s compared with the subthreshold membrane potential waveform. Again, spikes are not generated at the times of peak depolarization by the two-frequency stimulus. ( **F**) Spike timing when both stimuli are present, compared with the subthreshold membrane potential waveform in the presence of the 45 Hz sine wave alone. Note that, in each case, the neuron is phase-locked to the frequency component close to its own firing rate and there is little influence from the other stimulus frequency.

When multiple input frequencies are present simultaneously, a non-resonant neuron may respond specifically to a single input closest to the cell’s own firing rate, as illustrated in Figure 4. An input current composed of two oscillations at different frequencies produces a complex subthreshold membrane potential waveform. When the cell is depolarized and firing, it preferentially phase-locks to the stimulus oscillation close to its own firing rate, regardless of oscillatory inputs of the same amplitude but different frequency. When firing at a different rate, the cell shifts its firing to phase-lock with an input oscillation that it disregarded previously. Therefore, the frequency preference of the non-resonant neuron may be tuned by non-periodic inputs that adjust its average firing rate.

## Conclusions

Spiking in both resonant and non-resonant cells is entrained by oscillating inputs at specific frequencies. In resonant cells, the cells’ frequency preferences are relatively fixed, determined by the kinetics of their ion channels. In non-resonant cells, the frequencies of entrainment are tuned by the cells’ background firing rates, which in turn may be modulated by slower input components. Oscillatory inputs to neurons, like those that produce oscillations of the LFP, may not influence the firing of every neuron in the network but only a subset tuned to that frequency. Neurons with similar resonances (for example, cells resonant at gamma frequencies) may be entrained (and synchronized) by their shared resonant frequency, even when it is not prominent in their input (for example, 23, 34). This may contribute to the origin of gamma oscillations in the cortex ^42^.

Patterned population activity gives rise to the field potential, but most brain regions have more than one population of cells and more than one cell ensemble pattern expressed at any moment. These patterns are superimposed and confounded in the field potential. Understanding the structure of neuronal population activity requires a cellular and local circuit perspective.
